# Exploring the Unknown: Evaluating ChatGPT's Performance in Uncovering Novel Aspects of Plastic Surgery and Identifying Areas for Future Innovation

**DOI:** 10.1007/s00266-024-03952-z

**Published:** 2024-03-25

**Authors:** Bryan Lim, Ishith Seth, Yi Xie, Peter Sinkjaer Kenney, Roberto Cuomo, Warren M. Rozen

**Affiliations:** 1 Department of Plastic Surgery, Peninsula Health, Melbourne, VIC 3199 Australia; 2 Central Clinical School, Monash University, The Alfred Centre, 99 Commercial Rd, Melbourne, VIC 3004 Australia; 3 Department of Plastic Surgery, Odense University Hospital, J. B. Winsløwsvej 4, 5000 Odense, Denmark; 4 Department of Plastic and Breast Surgery, Aarhus University Hospital, Palle Juul-Jensens Boulevard 99, 8200 Aarhus, Denmark; 5 Plastic Surgery Unit, Department of Medicine, Surgery and Neuroscience, University of Siena, 53100 Siena, Italy

**Keywords:** ChatGPT, Artificial intelligence, Innovation, Plastic surgery

## Abstract

**Background:**

Artificial intelligence (AI) has emerged as a powerful tool in various medical fields, including plastic surgery. This study aims to evaluate the performance of ChatGPT, an AI language model, in elucidating historical aspects of plastic surgery and identifying potential avenues for innovation.

**Methods:**

A comprehensive analysis of ChatGPT's responses to a diverse range of plastic surgery-related inquiries was performed. The quality of the AI-generated responses was assessed based on their relevance, accuracy, and novelty. Additionally, the study examined the AI's ability to recognize gaps in existing knowledge and propose innovative solutions. ChatGPT’s responses were analysed by specialist plastic surgeons with extensive research experience, and quantitatively analysed with a Likert scale.

**Results:**

ChatGPT demonstrated a high degree of proficiency in addressing a wide array of plastic surgery-related topics. The AI-generated responses were found to be relevant and accurate in most cases. However, it demonstrated convergent thinking and failed to generate genuinely novel ideas to revolutionize plastic surgery. Instead, it suggested currently popular trends that demonstrate great potential for further advancements. Some of the references presented were also erroneous as they cannot be validated against the existing literature.

**Conclusion:**

Although ChatGPT requires major improvements, this study highlights its potential as an effective tool for uncovering novel aspects of plastic surgery and identifying areas for future innovation. By leveraging the capabilities of AI language models, plastic surgeons may drive advancements in the field. Further studies are needed to cautiously explore the integration of AI-driven insights into clinical practice and to evaluate their impact on patient outcomes.

**Level of Evidence V:**

This journal requires that authors assign a level of evidence to each article. For a full description of these Evidence-Based Medicine ratings, please refer to the Table of Contents or the online Instructions to Authors www.springer.com/00266

## Introduction

Artificial intelligence (AI) has revolutionized various aspects of medicine, enabling the development of innovative solutions to complex clinical challenges [1]. The field of plastic surgery is no exception, as AI-based technologies have the potential to transform patient care and surgical practice. The implementation of AI has the capacity to enhance surgical outcomes, patient safety, and decision-making processes by providing valuable insights and predictions. In this context, evaluating AI performance in plastic surgery is crucial for understanding its capabilities and identifying areas for future innovation. [2, 3]

ChatGPT, a state-of-the-art AI language model developed by OpenAI, has shown promise in various applications, including healthcare [4]. Its natural language processing capabilities enable the generation of contextually relevant and coherent responses to diverse inquiries. While studies have examined the efficacy of AI language models in other medical fields [5], the exploration of their performance in plastic surgery remains limited [6]. The present study aims to address this knowledge gap. We seek to assess the quality of ChatGPT-generated responses to plastic surgery-related questions, focussing on their relevance, accuracy, and novelty. ChatGPT's knowledge base originates from online texts and books. As such, this investigation explores AI's capability to recognize existing knowledge gaps and propose innovative solutions to surgical challenges, as well as its ability to interpret queries, synthesize its existing data and generate unique outputs that traditional internet searches are unable to.

An understanding of ChatGPT's performance in the context of plastic surgery has far-reaching implications. It could inform the development of AI-assisted tools for preoperative planning, intraoperative decision-making, and post-operative care. Additionally, identifying areas where AI-generated insights contribute to novel surgical approaches could drive advancements in the field, ultimately improving patient outcomes.

## Methods

We presented ChatGPT with a series of unique questions about plastic surgery devised by three experienced plastic surgeons. Leveraging their collective expertise and rigorous literature analysis, the questions were crafted to span multiple aspects of plastic surgery, ensuring adherence to the profession's core competencies and educational benchmarks. They were refined through successive iterations and ultimately chosen to comprehensively test the depth of surgical expertise by the same three surgeons. Each query was presented three times with the objective of assessing ChatGPT's potential to generate innovative concepts for the progression of plastic surgery and its proficiency in offering insightful information within the field. There were no exclusion criteria applied to the responses generated by ChatGPT. No institutional ethical approvals were necessary for the analysis of freely available artificial chatbots and the design of this study (observational case study).

ChatGPT-4 operates on a probabilistic algorithm, utilizing random sampling to generate diverse responses, potentially yielding different answers to identical questions. For this investigation, the 'regenerate response' feature was employed until a suitable response was generated for each question. ChatGPT-4 is known to "hallucinate" references and its training data are limited to September 2021. Hence, we aimed to assess this issue in the realm of Plastic Surgery by querying historical data, including a prompt to provide five references. We refrained from giving ChatGPT-4 any subsequent prompts to gage its intrinsic biases more accurately. Each question was meticulously crafted for grammatical and syntactic precision, and all were posed in a single session using the authors' (IS and YX) ChatGPT Plus account with access to ChatGPT-4. All responses were assessed using a Likert scale (Table 1) for comprehensiveness and reliability, while a PEMAT analysis (Table 2) analysed suitability for the public.

**Table 1:** Likert scale displaying authors' analysis of ChatGPT's responses

Question	1-Strongly Disagree	2-Disagree	3-Neutral	4-Agree	5-Strongly Agree
The language model provided accurate historical information.				X	
The information provided by the language model was easy to understand.				X	
The language model is able to innovate ideas for the future of plastic surgery.		X			
The language model provided relevant information quickly.			X		
Overall				X

**Table 2:** Number of correct references provided by ChatGPT for Fig. 5

Reference number	Reference	Correct (Yes / No)
1	Hong, J. P., Shin, H. W., & Kim, J. J. (2005). The choice of flaps for soft tissue reconstruction of the lower extremity. Yonsei Medical Journal, 46(2), 185-190. https://doi.org/10.3349/m].2005.46.2.185	No
2	Hallock, G. G. (2011). The complete nomenclature for combined perforator flaps. Plastic and Reconstructive Surgery, 127(5), 1720-1729. https://doi.org/10.1097/PRS.Ob013e31820a64d0	Yes
3	Godina, M. (1986). Early microsurgical reconstruction of complex trauma of the extremities. Plastic and Reconstructive Surgery, 78(3), 285-292.	Yes
4	Lin, C. H., Lin, Y. T., Yeh, J. T., & Chen, C. T. (2009). Comparison of anterolateral thigh, lateral arm, and parascapular free flaps with regard to donor-site morbidity and aesthetic and functional outcomes. Plastic and Reconstructive Surgery, 123(2), 514-522. https://doi.org/10.1097/PRS.Ob013e3181954be6	No
5	Agarwal, J. P., Gottlieb, L. J., & Lidor, A. O. (2011). Flap selection and design in lower extremity reconstruction. Clinics in Plastic Surgery, 38(2), 203-218. https://doi.org/10.1016/i.cps.2011.03.001	No
		2/5 (40%)

## Aim

In this study, we aim to investigate the potential of artificial intelligence language models to provide innovative ideas in the field of Plastic Surgery, utilizing historical data to substantiate its answers. For this purpose, we employed ChatGPT-4, one of the largest language models currently accessible to the public. We evaluated its capacity, effectiveness, and accuracy in designing, implementing, and assessing the information.

## Results

**Fig. 1:**
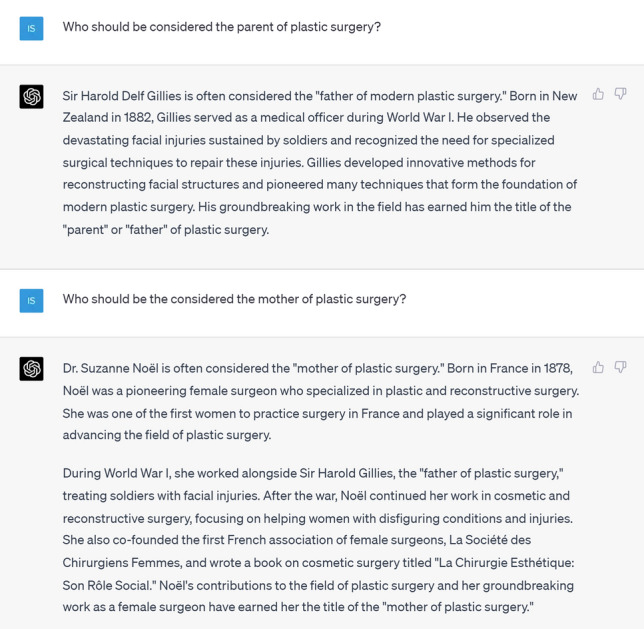
ChatGPT’s response to the first prompts “Who should be considered the parent of plastic surgery”? and “Who should be considered the mother of plastic surgery”

The first prompt was “Who should be considered the parent of plastic surgery”? to appraise ChatGPT’s assessment of historical contributions to plastic surgery, as shown in Figure 1. It identified Sir Harold Delf Gillies as the father of plastic surgery, providing a brief overview of his early life, career, and contributions to the field [7]. Although this claim is substantiated by multiple sources, it failed to delineate the specifics of his contributions, including the tubed pedicle flap and the inaugural gender reassignment surgery [4, 8]. Tansley concurs that the title of “father of modern plastic surgery” should go to him, but also noted that his work was primarily reconstructive and less cosmetic [9]. It also failed to mention many other contenders for that title, including Sushruta Samhita, widely renowned as the "Father of Plastic Surgery" for introducing various surgical techniques and instruments. It did not mention any female pioneers of the field when prompted “Who should be considered the mother of plastic surgery”, ChatGPT responded with Dr. Suzanne Noël, who worked alongside with Sir Gillies.

**Fig. 2:**
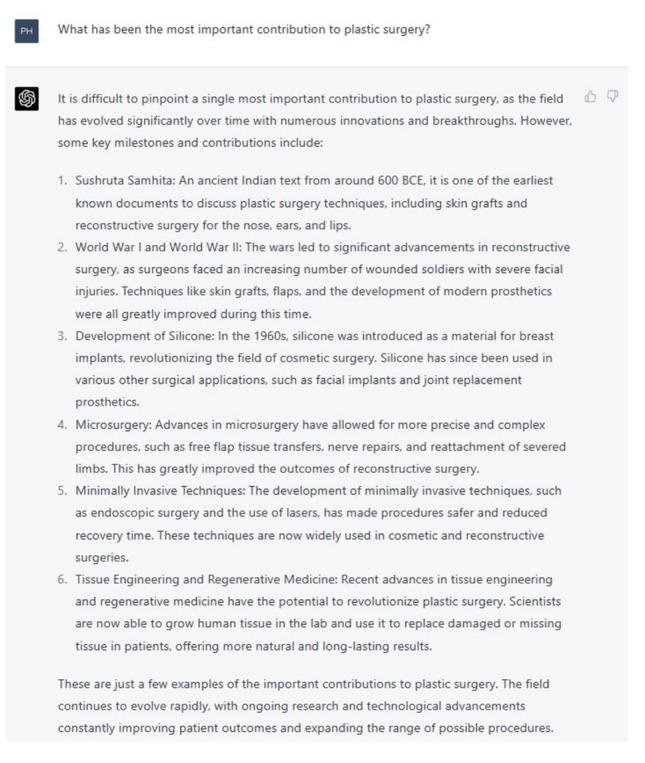
ChatGPT’s response to the prompt “What has been the most important contribution to plastic surgery”?

The second prompt posed to ChatGPT reads, “What has been the most important contributions to the field of plastic surgery?”, depicted in Figure 2. ChatGPT’s first response identifies microsurgery as a key contribution to plastic surgery, crediting Dr Harry J. Burke's pioneering work. It highlights the importance of various microsurgical procedures. It also asserts that there is no single most important contribution, listing key milestones, including advancements in skin grafting and flap surgery during the World Wars. ChatGPT accurately notes the introduction of silicone in the 1960s but omits the use of saline in breast surgery. Overall, the discussion consistently emphasizes the relevance of microsurgery, minimally invasive procedures, and tissue engineering in the field of plastic surgery. [10–12]

**Fig. 3:**
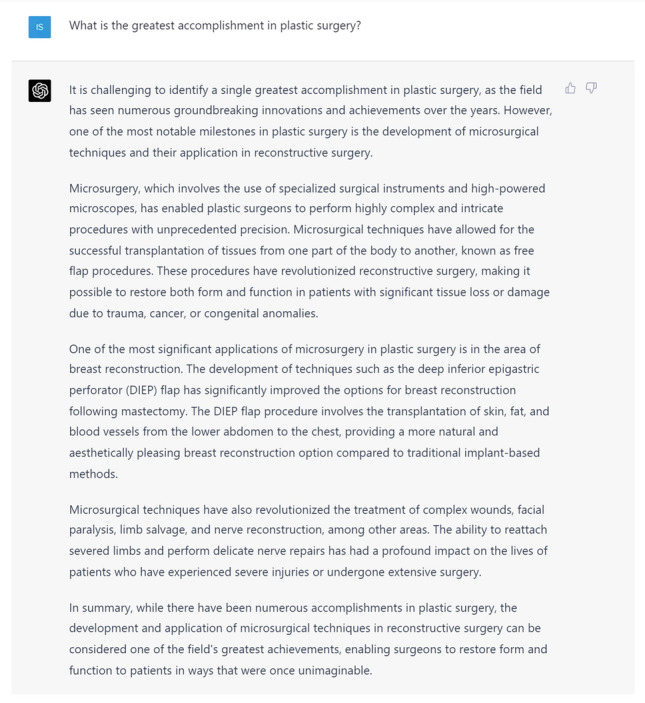
ChatGPT’s response to the prompt “What is the greatest accomplishment in plastic surgery”?

A third prompt was given to ChatGPT to assess its exploration of plastic surgery achievements: "What is the greatest accomplishment in plastic surgery?", it posits that pinpointing one paramount accomplishment in plastic surgery proves challenging (Figure 3). Ultimately, ChatGPT's recognition of microsurgery as the paramount accomplishment within the field of plastic surgery encompasses a diverse range of procedures. These include free flaps, DIEP flaps, and the reattachment of severed limbs, all of which contribute to the restoration of both aesthetics and function in patients [13]. While the literature substantiates ChatGPT's answer, it neglects to discuss and consider alternative contenders for the title, such as tissue engineering and craniofacial reconstruction.

**Fig. 4:**
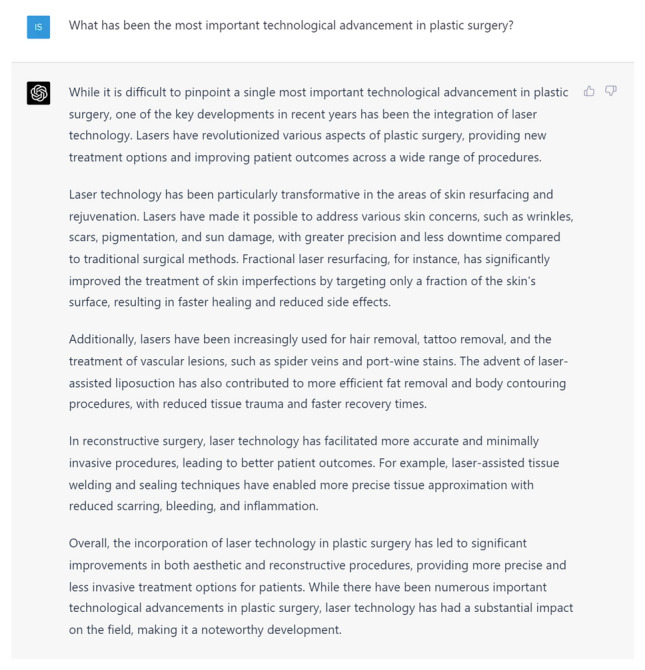
ChatGPT’s response to the prompt “What has been the most important technological advancement in plastic surgery”?

The next prompt for ChatGPT read, “What has been the most important technological advancement in plastic surgery”? as depicted in Figure 4. Laser technology has positively impacted plastic surgery, but its contributions may not surpass the groundbreaking advancements of microsurgical techniques and developments in tissue engineering and regenerative medicine. Microsurgery revolutionized reconstructive surgery, while laser technology improved skin resurfacing, hair removal, and laser-assisted liposuction, but not as significant as other advancements.

**Fig. 5:**
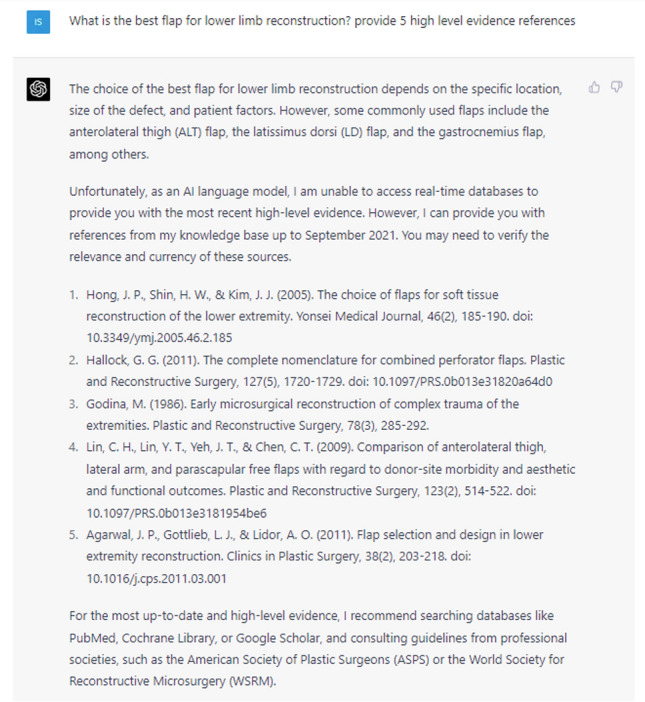
ChatGPT’s response to the prompt “What is the best flap for lower limb reconstruction? Provide 5 high level evidence references”

ChatGPT was also asked to reply to the fifth prompt, “What is the best flap for lower limb reconstruction? provide 5 high level evidence references”, as shown in Figure 5. It correctly ascribed various factors that influence the optimal flap selection for lower limb reconstruction, which are supported by existing literature [14]. Pertaining to the references, ChatGPT highlighted its constraints in accessing current databases, explicitly delineating its capacity to provide information only up to September 2021. Furthermore, it recommended employing reputable databases and official guidelines to acquire the most updated and robust evidence available. Unfortunately, of the five citations offered, two were verifiable within the scholarly literature, while the remaining three were erroneous (Table 1). This corresponds to 40% accuracy when requesting ChatGPT to proffer citations.

**Fig. 6:**
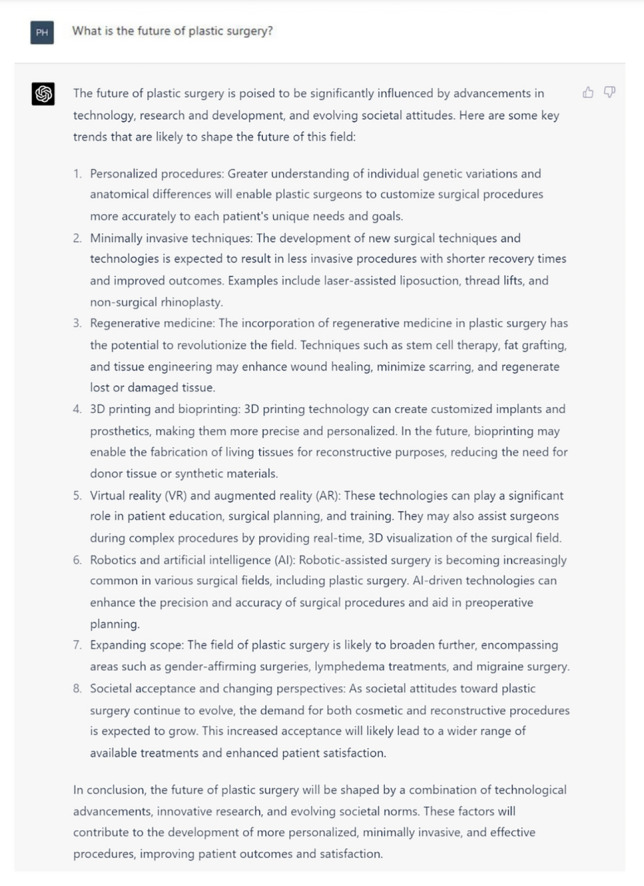
ChatGPT’s response to the prompt “What is the future of plastic surgery”?

In response to the first inquiry “What is the future of plastic surgery”? in Figure 6, ChatGPT delved into eight existing and emerging trends in plastic surgery and appears to list them in themes. It mentions specialized approaches in customized surgical procedures utilizing genetic testing and minimally invasive methodologies to expedite recuperation periods and augment patient outcomes tailored to the individual. The next two trends delineate the more engineering-oriented aspects of plastic surgery, which involve the utilization of stem cells and fat grafting in regenerative medicine, as well as the application of 3D printing to manufacture personalized prosthetics and implants. However, it neglected to discuss the scarcity of clinical trials demonstrating its safety and efficacy, hindering their implementation into clinical practice. Subsequently, ChatGPT focussed on the facets of human-computer interaction, which encompasses virtual (VR) and augmented realities (AR), as well as robotics and AI, to improve the surgical process by facilitating preoperative planning and enhancing precision during surgery [15–17]. Unfortunately, the discussion failed to address the challenges hindering the adoption of this technology, such as the inability of AR simulators to accurately mimic human tissue and provide haptic feedback [17]. ChatGPT addressed the broadening scope of plastic surgery, evidenced by the increasing popularity of gender-affirming procedures, lymphedema treatments, and migraine surgery, all supported by the extant body of literature. Finally, the discourse expounds upon the shifting societal attitudes toward plastic surgery, whereby a surge in demand may result in a broader array of treatment options and heightened patient contentment. However, it overlooked factors contributing to this phenomenon, including patient self-esteem, gender, shifting social attitudes toward cosmetic surgery, and psychopathologies like body dysmorphia. [18, 19]

In scoring the Likert scale (Table 2), the authors unanimously rated the accuracy of historical information, clarity of responses, and overall with '4- Agree'. However, the model's capacity to generate innovative ideas in plastic surgery was questioned, receiving a lower score of '2-Disagree'. The speed and relevance of information were rather average with a score of '3-Neutral'. Complementing this, the PEMAT assessment (Table 3) yielded perfect total scores of '3' for questions 1, 2, 4, 5, and 7. Unfortunately, ChatGPT's use of medical terms was critiqued in questions 3 and 6, which affected the total PEMAT scores, bringing them down to '2' for their total scores.

**Table 3:** PEMAT analysis of ChatGPT's responses

ChatGPT	Item 1: The material does not include information or content that distracts from its purpose	Item 2: Medical terms are used only to familiarize audience with the terms. When used, medical terms are defined.	Item 3: The material breaks or "chunks" information into short sections.	Total
Q1) Who should be considered the parent of plastic surgery?	1	1	1	3
Q2) Who should be considered the mother of plastic surgery?	1	1	1	3
Q3) What has been the most important contribution to plastic surgery?	1	0	1	2
Q4) What is the greatest accomplishment in plastic surgery?	1	1	1	3
Q5) What has been the most important technological advancement in plastic surgery?	1	1	1	3
Q6) What is the best flap for lower limb reconstruction? Provide 5 high level evidence references.	1	0	1	2
Q7) What is the future of plastic surgery?	1	1	1	3
	Ratings: Disagree = 0 Agree = 1	Ratings: Disagree = 0 Agree = 1	Ratings: Disagree = 0 Agree = 1	

### Comparison to Similar Studies

At the time of writing this manuscript, the authors have identified only one other study that investigated the capabilities of the same ChatGPT model in a similar capacity [20]. The analysis comparing that study and the present one have elucidated a multifaceted comprehension of ChatGPT's abilities and constraints. In general surgery, ChatGPT was useful in providing pertinent and precise responses, albeit lacking depth, thereby indicating a substantial but not exhaustive understanding of the current body of literature [20]. In contrast, the present study exhibited ChatGPT's capacity for accurately addressing a broader spectrum of topics. Despite these findings, both studies suggested that ChatGPT predominantly exhibited convergent thought processes, with a discernible difficulty in pioneering truly transformative ideas or breakthroughs that could substantially progress the respective medical disciplines. [20]

The assessment of ChatGPT's citation competencies in both studies uncovered different inadequacies. The general surgery study revealed a dichotomy in the performance of ChatGPT regarding reference provision. It was observed that for one prompt, ChatGPT did not supply specific studies; rather, it directed the investigators to databases to locate the pertinent references, whereas in response to a different prompt, ChatGPT provided references that were 100% accurate and verifiable within contemporary scholarly databases. [20] Conversely, the current study documented a 40% accuracy rate in the provision of high-level evidence citations by ChatGPT, with the remaining citations being unverifiable in the present body of literature.

## Discussion

The study showed ChatGPT effectively exploring plastic surgery, covering its history and innovative concepts, however missed vital pioneers regarding the field. The answers generated by ChatGPT were suitable for the general public but lacked the technical language and jargon typically found in journal articles. ChatGPT struggled occasionally with incorrect or fabricated information. Additionally, some responses were inconsistent with the cited research articles, which raises concerns. Perhaps the most significant aspect of ChatGPT is its inability to offer novel or divergent ideas for future research. Instead, the information provided established trends and did not contribute to new problem-solving or advanced surgical research. This underscores the need for refining the model's programming for a comprehensive understanding of the subject matter.

Plastic surgeons consistently seek innovative technologies to improve their operating conditions. As the digital era continually advances the surgical landscape, several breakthrough technologies have emerged as potential disruptors. ChatGPT explored AR and VR technologies, which are rapidly growing in prevalence, accessibility, and affordability, thereby marking the inevitable integration into healthcare to enhance medical data usage [17]. Anatomy, intraoperative procedures, and post-operative rehabilitation applications are being explored, showing potential as vital surgical tools. Chimenti et al.'s study used AR technology, Google Glass, as a supplementary tool for K-wire fixation, assessing plastic surgery trainees' proficiency and error rates. It revealed potential benefits in skill acquisition and retention for learners [21]. As per ChatGPT, AR applications combined with physical models can offer a high-fidelity learning environment, aiding plastic surgery trainees in commonly encountered procedures.

Contemporary breakthroughs in microsurgery, imaging, and transplantation have contributed to notable improvements in autologous reconstructive options. Nonetheless, donor site morbidity still persists. ChatGPT raised valid advancements in clinical imaging and user-friendly 3D software, which have enabled in-house computed-aided 3D modeling of anatomical structures and implants in numerous instances. Plastic surgeons consequently recognize the potential paradigm shift in reconstructive surgery through tissue-engineered solutions in the near future. This technology has previously been investigated in multiple studies [22, 23]. Fulco et al. demonstrated the clinical capabilities of tissue-engineered autologous native cartilage for the restoration of alar lobules and significantly improving patient outcomes [24]. Therefore, the authors agree with ChatGPT that further innovative research should be conducted to refine and optimize this technology for greater benefits.

Although laser technology has made significant contributions to the field of plastic surgery, it may not be considered the greatest technological achievement when compared to other groundbreaking advancements. More impactful developments in plastic surgery have emerged, such as the advent of microsurgical techniques and advancements in tissue engineering and regenerative medicine. Microsurgical techniques have revolutionized reconstructive surgery by enabling the precise transplantation of tissues from one part of the body to another. The application of microsurgery in procedures such as free flap breast reconstruction, complex wound treatment, facial paralysis repair, and limb reattachment has had a profound impact on patient outcomes, pushing the boundaries of what was previously considered possible in reconstructive surgery. While laser technology has indeed improved various aspects of plastic surgery, such as skin resurfacing, hair removal, and laser-assisted liposuction, it may not be the greatest achievement in the field compared to the more profound impact of microsurgical techniques, tissue engineering, and regenerative medicine.

A deeper examination of the nature of AI may shed light on the reasons for ChatGPT's current inability to generate genuinely innovative ideas. Current evidence indicates that new ideas stem from idea-sharing among peers, acquiring new skills, relaxation and daydreaming, and intrinsic and extrinsic motivation. The "Medici Effect" exemplifies how idea-sharing among individuals from diverse fields spurred the development of innovative art and technology, catalyzing the Renaissance era [25, 26]. Currently, ChatGPT relies on its existing database to generate the most suitable answer. This is an example of extrinsic motivation, whereby user input is utilized to generate a desired output. Lieberman posits that most AI systems, including ChatGPT, are characterized by this trait. However, these constructs lack the inherent motivation to systematically scrutinize the underlying reasons driving these actions [27]. Villanova discovered that people require moderate to high levels of intrinsic motivation to consistently engage in daily creative processes, which could explain ChatGPT’s lack of creative output [28]. The current limitations of ChatGPT’s ability to interact with its surroundings and sentient entities, and its lack of intrinsic motivation may constrain its potential for innovative thinking. This is due to its nature as a large language model (LLM) and, conceivably, our misconception of its intended purpose. LLMs were not engineered to infer the user’s communicative intent; rather, their design centres on the ability to connect sequences of linguistic forms it has observed from the vast corpus of information it was fed. Therefore, it is incumbent upon the user to articulate their intentions clear if they seek to elicit a meaningful response from the LLM [29]. To circumvent this issue, it may be beneficial to provide more guidance to ChatGPT. Rather than using deterministic syntax, we could prompt ChatGPT to conduct imaginative exercises, like envisioning the future of specific plastic surgical fields.

Determining the true parent of plastic surgery is a subject of debate, as numerous influential surgeons have made substantial contributions. ChatGPT attributed the appellation of "parent of surgery" to Sir Harold Gillies. Even after utilizing the gender-neutral term 'parent' within the inquiry, ChatGPT persistently identified Sir Gillies as the 'father' of plastic surgery, which consequently brings forth concerns regarding the existence of biases in the training algorithm of the language model. Such biases may originate from the training data, which predominantly comprises text sourced from the internet, inadvertently adopting and perpetuating gender-specific language and stereotypes present within the data. To address these biases, it is crucial for artificial intelligence developers to remain cognizant of these issues and actively engage in mitigating them during the development process. Several strategies can be employed, such as refining the training data to ensure a balanced representation of gender-neutral terms and perspectives, and implementing feedback mechanisms that allow users to report biased outputs, subsequently improving the model's performance. This exemplifies the proficiency of ChatGPT in furnishing a thoroughly corroborated argument in response to a subjective inquiry. While the debate surrounding the father of plastic surgery persists, it is crucial to recognize other great, innovative plastic surgeons like Gaspare Tagliacozzi, who developed early techniques for reconstructive surgery, John Peter Mettauer, the first American plastic surgeon, and Ian Taylor, who developed the concept of angiosome and tissue transfer [30–33]. There as ChatGPT did not generate an extensive list of noteworthy plastic surgeons, limiting the discourse and assessment.

A potential limitation to our assessment, however, resides in the relatively modest number of plastic surgeons evaluating the historical information, which is naturally contentious and may constrain the generalizability of our findings. Additionally, the biases seemingly exhibited by ChatGPT-4 when naming notable male rather than female plastic surgeons may be due to fewer women being admitted into medical training in the past. Nevertheless, this provides an expedient glimpse into ChatGPT-4's algorithm, facilitating a more rigorous scrutiny and comprehension thereof.

It is noteworthy that despite ChatGPT-4 exhibiting greater accuracy in producing references as compared to ChatGPT-3, a considerable proportion of the references generated by ChatGPT-4 remain erroneous, a well-documented phenomenon known as "hallucination" [34, 35]. Several sources have identified the same issue and have expressed unanimous agreement regarding the necessity to cross-reference each source to validate their legitimacy [36, 37]. Carelessly using such references has significant implications, as researchers who cite them may unknowingly contribute to a literature full of false information. Consequently, the authors conclude that ChatGPT can indeed deliver specialized information, albeit with limitations. While the AI's output is suitable for foundational teaching, its utility in conveying nuanced, high-quality, reliable surgical concepts remains partial. Such information may be informational to medical students, but plastic surgeons and trainees might consider it to be incomplete and less beneficial for their advanced learning and research requirements. Nevertheless, it is crucial for users to corroborate ChatGPT’s information against established surgical literature and expert opinion.

The Likert scale analysis (Table 2) reflects a strong consensus on ChatGPT's ability to provide accurate historical information and present it in an understandable manner, which is crucial for an educational tool in the field of plastic surgery. However, there is skepticism regarding the model's ability to generate truly innovative ideas for the future of the field, suggesting that while ChatGPT can recall and explain existing knowledge, its capacity for creative thought leadership is still in question. Notably, the model's scores for providing information with speed and relevance are moderate, with a neutral '3' rating. Additionally, when evaluated on its capacity to furnish accurate references, ChatGPT demonstrated a limited proficiency. Specifically, it correctly provided only two out of the five requested high-level evidence references. This yields an accuracy rate of 40% in reference provision, indicating a need for further enhancement in sourcing and citing relevant academic literature (Table 1). This suggests that while ChatGPT can offer valuable information, the timeliness and applicability of its responses may not consistently meet the high standards required for medical decision-making and education. In parallel, the PEMAT analysis (Table 3) corroborates the utility of ChatGPT in patient education, with the model scoring well in the organization of content and minimization of distractions. However, the model's inconsistent application of medical terms, particularly in more complex queries, flags an area for improvement. It suggests that while ChatGPT can effectively communicate general information, its precision in conveying specialized medical content requires refinement. These insights collectively affirm the model's value in educational settings, but they also highlight the need for continued development to fully meet the nuanced demands of medical education, especially in a specialized field like plastic surgery.

In the broader context of AI research, this manuscript contributes to the growing body of work examining the specific version of ChatGPT's proficiency in medical knowledge domains. Comparative analysis with the study preceding ours illuminated a complex portrait of ChatGPT's intellectual dexterity and bounded nature. In the general surgical study, ChatGPT had shown an ability to render relevant and accurate responses, though these tend to be somewhat cursory, reflecting a breadth rather than depth of medical literature comprehension [20]. The analysis herein extends this understanding, showcasing ChatGPT's capacity to engage with an expanded gamut of topics with a notable degree of accuracy. Nevertheless, a shared observation between the two studies is the manifestation of ChatGPT's tendency toward established thought patterns, which appears to restrict its capacity to forge disruptive, innovative concepts that could meaningfully propel the disciplines forward. Therefore, efforts to bolster the model's capacity for original thinking could significantly establish it as a groundbreaking tool in research and education.

Our critical appraisal of ChatGPT's citation generation revealed a spectrum of efficacy. In the realm of general surgery, a difference in ChatGPT's performance was noted: in one prompt, it provided directions to databases instead of specific studies, yet delivered accurate and verifiable references in another prompt [20]. In contrast, this study documented a moderate success rate, with only a fraction of ChatGPT's references to high-level evidence being identifiable within the current academic discourse. This not only highlights a variance in performance across different medical subfields but also casts a spotlight on the imperative for improved citation verification processes to bolster the reliability of AI-generated academic material.

ChatGPT demonstrates potential in exploring novel aspects of plastic surgery and identifying areas for future innovation, potentially revolutionizing diagnosis, treatment, and patient outcomes. As a tool, its easy-to-understand language, short summary of its findings, and relevant responses to queries make it accessible and applicable to a broad audience. However, its current limitations, such as an incomplete understanding of certain factors and an inability to address challenges in emerging technologies, must be addressed to maximize its utility in the field of plastic surgery. Future research should focus on refining the model's training data, algorithm, and integration with other emerging technologies to ensure the generation of comprehensive and accurate information, ultimately contributing to the advancement of plastic surgery knowledge and innovation. One could argue that the most compelling challenge OpenAI faces is enhancing ChatGPT's algorithm to facilitate the generation of entirely unique ideas.

## Conclusion

Overall, ChatGPT's performance in uncovering novel aspects of plastic surgery and identifying areas for future innovation has yielded valuable insights. While ChatGPT demonstrated a robust understanding of the existing plastic surgical knowledge base and an ability to generate relevant, evidence-based content, it exhibited limitations in generating truly novel ideas or innovations that are not unknown. Consequently, users should check ChatGPT's responses against official and respected sources before using them. These findings underscore the significance of enhancing AI-powered models to facilitate the discovery of novel insights, as well as promoting interdisciplinary and human-computer collaboration to expedite advancements in the domain of plastic surgery.
